# A Review of Olanzapine in the Treatment of Cancer Anorexia-Cachexia Syndrome

**DOI:** 10.3390/pharmacy12010034

**Published:** 2024-02-17

**Authors:** Ivy O. Poon, Veronica Ajewole, Ursula K. Braun

**Affiliations:** 1 Department of Pharmacy Practice, Texas Southern University, Houston, TX 77004, USA; ivy.poon@tsu.edu (I.O.P.); veronica.ajewole@tsu.edu (V.A.); 2 Pharmacy Department, Houston Methodist Hospital, Houston, TX 77004, USA; 3 Rehabilitation & Extended Care Line, Michael E. DeBakey VA Medical Center, Houston, TX 77030, USA; 4 Section of Geriatrics & Palliative Medicine, Baylor College of Medicine, Houston, TX 77030, USA

**Keywords:** olanzapine, neoplasms, anorexia, cachexia

## Abstract

Background: Cancer anorexia-cachexia syndrome (CAS) is a multifactorial condition that is highly prevalent in advanced cancer patients and associated with significant reduction in functional performance, reduction in quality of life, and increased mortality. Currently, no medications are approved for this indication. Recently, the American Society of Clinical Oncology (ASCO) released a rapid recommendation suggesting that low-dose olanzapine once daily may be used to treat cancer cachexia. Many questions still exist on how to use olanzapine for this indication in clinical practice. The objective of this review is to identify existing knowledge on the use of olanzapine for CAS. Methods: A comprehensive search was conducted to identify the primary literature that involved olanzapine for anorexia and cachexia in cancer patients between 2000 and 2023. Results: Seven articles were identified and are discussed here, including two randomized double-blinded placebo-controlled studies, one randomized comparative study, two prospective open-label studies, one retrospective chart review, and one case report. Conclusions: Low dose olanzapine (2.5–5 mg once daily) may be useful in the treatment of CAS for increasing appetite, reducing nausea and vomiting, and promoting weight gain. Further large-scale multi-center randomized placebo-controlled studies will be needed to investigate the impact of olanzapine on weight change in CAS patients.

## 1. Introduction

Cancer anorexia-cachexia syndrome (CAS) occurs in up to 70–80% of lung, gastric, and pancreatic cancer patients [1,2]. CAS is a complex multifactorial syndrome with ongoing skeletal muscle loss despite nutritional intervention, and the patient may or may not present with fat mass loss [3]. It is a significant clinical finding of great importance because the presence of cachexia is associated with shorter survival [4,5,6], poor physical functioning [7], reduced response to chemotherapy, and a negative impact on the quality of life [8]. Refractory cachexia in advanced non-curable cancer patients has a prognosis of less than 3 months [9]. The presentation of CAS begins with the pre-cachexia stage, in which the patient presents with weight loss ≤ 5%, anorexia, and hypermetabolism. Cancer-induced anorexia is defined as reduced appetite and food intake secondary to cancer [10]. A patient may progress from pre-cachexic to cachexia. The diagnostic criteria of cancer cachexia have been suggested by an international expert panel to be those with cancer who experience a weight loss of >5%, those who have a BMI < 20 and a weight loss of >2%, or those who have sarcopenia and a weight loss of >2% [3]. Sarcopenia is defined as low muscle strength, low muscle quantity or quality, and low physical performance [11]. Clinical symptoms of CAS include appetite loss, nausea, glucose intolerance, and unpleasant changes in taste and smell [12]. 

The pathogenesis of CAS is complex and only partially understood [13]. The main cause is thought to be due to increased inflammatory cytokines Interleukin-1 (IL-1), IL-6, and Tumor Necrosis Factor- (TNF-) Alpha in cancer patients. Leptin is a peripheral hormone that regulates food intake and energy expenditure. An increase in leptin is associated with reduced appetite and food intake [13]. Interestingly, leptin has been reported to be reduced in CAS patients compared to those without CAS, which implied that CAS patients might be resistant to leptin’s regulation, a complex phenomenon related to inflammatory cytokines [1]. Patients with CAS are in a catabolic state with protein breakdown, a reduction in the body’s response to leptin and ghrelin signals, and increased total body expenditure [12]. Continuous assessments on weight change and anorexia, as well as muscle mass and strength, should be an integral part of the palliative symptom assessment and management for cancer patients [14]. The quality of life of the patient can be assessed by utilizing the functional assessment of anorexia/cachexia therapy (FAACT) scale (Cronbach’s alpha coefficient 0.9) [15].

Currently, no drug has FDA approval for the treatment of CAS, despite the significant morbidities and mortality. Short-term progesterone analog and corticosteroids have been suggested by the American Society of Clinical Oncology (ASCO) guideline with an intermediate evidence quality and moderate strength of recommendation [16]. In July of 2023, a rapid recommendation update of the ASCO guideline for the management of cancer cachexia was released, stating that low-dose olanzapine can be offered to improve appetite and weight gain in advanced cancer patients, while short-term progesterone analog or corticosteroids can be tried if the patient cannot tolerate olanzapine [17]. The guideline also stated that there is not enough evidence to suggest using mirtazapine for cancer cachexia based on clinical studies [18,19]. Now that low-dose olanzapine has become the first-line option for cancer cachexia, while the ACSO recommendation is very succinct, clinicians may ask for whom, how, and when to use low-dose olanzapine for CAS. The rationale of this review is to provide detailed information about the current published primary literature to inform clinicians of the best practices and examples in using olanzapine for this emerging off-label indication.

Olanzapine is a second-generation thienobenzodiazepine antipsychotic that has FDA-approved indications for the treatment of schizophrenia and acute manic or mixed episodes of bipolar disorder [20]. It acts as an antagonist of dopamine D1–4 and serotonin 5-HT3 receptors to produce the antipsychotic action [20]. It has a high affinity for 5-HT2A antagonism, resulting in its antiemetic action [21]. It is a strong antihistamine at the H1 receptor and causes sedation, which can be beneficial for treating insomnia [21]. Its action on H1, 5-HT2c, 5-HT2B, and D2 receptors has been reported to be associated with appetite stimulation effects [22]. It is also available in combination with fluoxetine for the treatment of resistant major depressive disorder. Oral olanzapine has a bioavailability of 60%. It undergoes direct glucuronidation to produce N-desmethylolanzapine, an inactive metabolite [23]. The half-time of oral olanzapine in adults is about 30 h, and older adults may have a longer half-life [23]. The long half-lives can be an advantage, allowing once-daily dosing to exert its action throughout the day without a need for multiple daily doses. Olanzapine is metabolized via the Cytochrome P450 2C9 and 2C6 pathway; those who are ultrarapid metabolizers (“UM”) may experience diminished response to the drug, and those with poor metabolism (“PM”) may experience an increased risk of extrapyramidal side effects [24]. In the U.S., oral olanzapine is available in generic formulations at a fraction of the brand-name cost.

## 2. Methods

**Figure 1:**
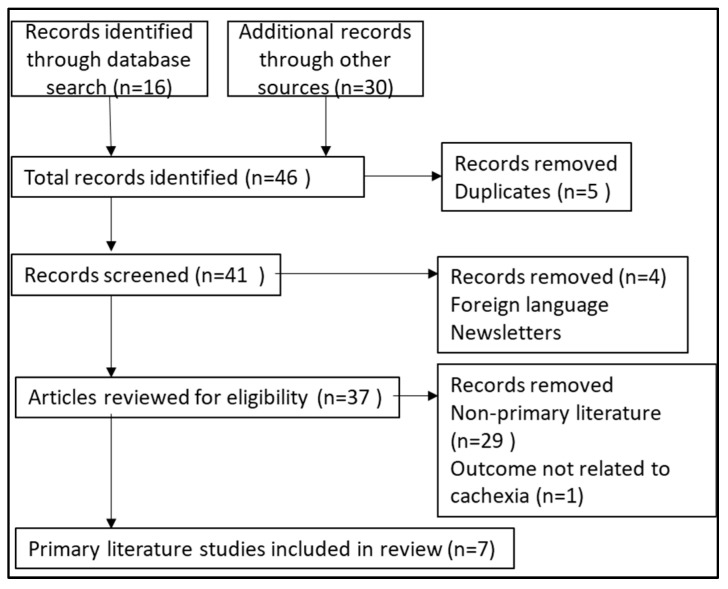
Algorithm for literature search and selection.

The objective of the literature search was to identify the existing primary literature (original research articles, reports, conference papers) describing the use of olanzapine for cancer cachexia. The MESH search terms cancer, cachexia, anorexia, and olanzapine were used to search PubMed, Cochrane Library, Medline (Ovid), Scopus, Embase, ClinicalKey, and CINAHL Plus databases. The search terms could be mentioned in the articles’ title, abstract, or body from 2000 to 2023. The search terms were entered into each database using the Boolean operator ‘AND’ in two combinations: ‘olanzapine’ AND ‘cachexia’ AND ‘cancer or ‘olanzapine’ AND ‘anorexia’ AND ‘cancer’. Additional searches were conducted through Google Scholar, Palliative Care Network websites [25], and the clinical trial registry [26]. This literature search was conducted from October to November of 2023. Search results were imported into the reference manager Zotero 6.0 [computer software] [27]. Figure 1 shows a search flowchart describing how articles were chosen for inclusion in this review [28]. There was no restriction on article types screened, but only the primary literature (e.g., clinical trials, retrospective studies, case reports) was eligible to be reviewed. Each article’s title and abstract were reviewed for relevance using the Zotero group files. One article was written in a foreign language and was excluded [29].

## 3. Results

Our review yielded seven original studies that fit into the eligibility criteria of primary literature studies on olanzapine use in CAS [30,31,32,33,34,35,36]. Among the seven studies, there were two randomized double-blinded placebo-controlled studies, one randomized comparative study, two prospective open-label studies, one retrospective chart review, and one case report. Two of those seven studies were published in both abstracts [37,38] and full articles [32,33]. Table 1 shows the details of the study design, outcomes, and results.

The first pilot study was conducted by Braiteh F. et al. at the MD Anderson Cancer Center in Houston, Texas, in 2008 [30]. The study investigated the effect and tolerability of olanzapine in doses of 2.5, 5, and 7.5 mg in 14 patients with advanced cancer. The therapeutic outcomes were measured in weight change, mini-nutritional assessment, Karnofsky performance scale (KPS), 6-min walking test, Edmonton Symptom Assessment Scale, inflammatory cytokines labs, and metabolic markers labs. The investigator reported an improvement in weight among 6 out of 14 participants in two weeks. Overall, improvements were observed in functional status measured in KPS and symptoms reported in ESAS, including sleep, appetite, and well-being. One limitation of this pilot study was a lack of control group and small sample size, but this study provided a positive result for further investigation on the use of olanzapine for this indication. Additionally, we are not able to find the full publication of this study; the findings above are based on an abstract publication.

The positive impact of olanzapine on anorexic cancer patients was also reported in a randomized study conducted by Narari et al. to investigate the effects of megestrol acetate with olanzapine compared to megestrol acetate alone in the Walther Cancer Research Center in Indiana [31]. This research study recruited adult patients with stage III-IV gastrointestinal or lung cancer, anorexia, and weight loss of ≥5% from pre-cancer stable weight. The study had an extensive list of exclusion criteria, including but not limited to no major surgery, radiotherapy, chemotherapy, or systemic corticosteroids within the past 4 weeks, uncontrolled diabetes, and having contraindications to the study medications. Eighty participants were randomly assigned to receive 800 mg/day of megestrol (*n* = 40) or 800 mg/day of megestrol plus 5 mg/day of olanzapine (*n* = 40) and monitored weekly for 8 weeks. The median age of the cohort was 63 years old (range, 39–81). This was not an intention-to-treat study, and those who were lost to follow-up or non-adherent to the regimen for 48 h were excluded in the final analysis (4 out of 80). Outcomes were measured in weight, patient-reported appetite, and nausea levels using a visual analog scale, as well as the M.D. Anderson Symptom Inventory score [39]. Investigators reported that 33 out of 39 patients who received megestrol plus olanzapine for 8 weeks experienced a >5% weight gain compared to 15 out of 37 patients in the megestrol-only group. Patients in the megestrol plus olanzapine group also reported significant improvement in general activity, mood, work, walking, enjoyment, appetite, and nausea compared to baseline (*p* < 0.01). One limitation of this study was that the investigational drugs were not blinded to the patients and investigators, who had the potential of introducing bias in the self-reported outcomes; however, weight is an objective measure, and this study’s finding showed a very promising improvement in weight in the megestrol plus olanzapine group. The study also added evidence that using low-dose olanzapine, without the need for dose titration, could be a potential strategy when administering olanzapine for this indication.

In 2015, Naing et al. reported a prospective open-labeled study of 39 patients with cancer-related cachexia [32]. The study recruited those with advanced cancer and a 10% weight loss within the past 6 months who were able to take oral medications, had an Eastern Cooperative Oncology Group (ECOG) performance status between 0 and 2, and had an expected life span of at least 3 months. The setting was at M.D. Anderson Cancer Center, a large academic research center in Houston, Texas. The study used a 3 + 3 dose escalation design, starting from an initial dose of 2.5 mg of olanzapine every other day to 20 mg daily. A total of 39 participants were enrolled, with 79% having an ECOG performance status of 1. Participants had a variety of cancers, including 31% colorectal, 13% pancreatic, 10% ovarian, 8% breast, 5% each for lung and renal, and 28% other cancers. Nine out of the thirty-nine patients were started on 20 mg of olanzapine daily, and only one experienced grade II extrapyramidal symptoms. Therefore, the authors concluded that 20 mg of olanzapine was tolerated by their cohort of advanced cancer patients. There was no preset duration of therapy; each participant continued the drug unless a grade 3 or higher adverse event occurred, or the patient or provider decided to stop the agent based on benefit and risk. Thirty-one out of thirty-nine participants were evaluable for the primary outcome (weight slope). The investigators observed only a modest effect on the rate of weight loss before and during the study and found a lack of correlation between inflammatory cytokines and weight change. One limitation of this study was the small sample size and the fact that cytokines levels might fluctuate depending on the different cancer stages, tumor type, and chemotherapy. However, this study provided evidence that 20 mg of olanzapine per day (dose for psychiatric indication) could be tolerated similarly in advanced cancer patients. Another limitation was that the study cohort had a median age of 57 (range 28–84); the tolerability of 20 g of olanzapine per day might not be generalized to the older adult population because olanzapine has been associated with increased risk for adverse drug events and mortality in older adults [40].

Sandhya et al. reported the first randomized, double-blind, placebo-controlled study investigating if 2.5 mg of olanzapine daily plus nutritional advice increased weight in a cohort of newly diagnosed advanced lung, gastric, and hepatopancreatic biliary (HPB) cancer patients on chemotherapy [33]. The setting was an academic research institute in India. The study recruited adult patients who were newly diagnosed with advanced lung, gastric, or HPB cancer and planned for the first cycle of chemotherapy, with ECOG performance status of 0 to 3 and the ability to take oral medications. Participants were randomly assigned to receive 2.5 mg of olanzapine daily (*n* = 63) or a placebo (*n* = 61) for 12 weeks. The study arm allocation was blinded to participants, clinicians, and nutritionists. Enrolled participants also received an individualized diet sheet emphasizing a high-protein, high-calorie, and nutritionally dense diet. Eighty-four percent of the participants had stage IV cancer. Participants were assessed on weight, Functional Assessment of Chronic Illness Therapy system of Quality-of-Life questionnaires for Anorexia Cachexia (FAACT ACS), nutrition status, chemotherapy toxicity, quality of life, and appetite using a visual analog scale. Among the 124 individuals allocated to each arm, 112 completed the study at 12 weeks, and their results were analyzed. The median age of the cohort was 55 (range, 24–74). The study was not intention-to-treat. The investigators reported that 35 out of 58 individuals in the olanzapine had a >5% increase in weight from baseline, versus only 5 out of 54 individuals in the placebo group. More individuals in the olanzapine group reported an improvement in appetite, calorie intake, and quality of life compared to the placebo group (*p* < 0.001, *p* = 0.0001, and *p* = 0.003, respectively). One limitation of this study was that the race and ethnicity of participants were not reported. Since the study was conducted in India, most participants were likely Asian Indians, and the generalizability to all population groups is unknown. Olanzapine is metabolized through the CYP450 2D6 pathway and genetic polymorphism variations among different racial and ethnic groups may exist, which will affect drug response and toxicity [24]. The average BMI was 20.6–20.7 at baseline in this study, with 35% underweight (BMI < 18.5). A study by Haro ML et al. reported that those with BMI < 21 experienced a greater weight increase compared to those who a higher BMI at baseline after using olanzapine for 6 months [41]. Therefore, the effects on weight may be less profound for those with a higher BMI at baseline. Additionally, this study focused on newly diagnosed cancer patients on chemotherapy, and having cachexia was not part of the inclusion criteria (85–94% self-reported anorectic at baseline); it is unknown if the results would implicate those who had cachexia and refractory cachexia without chemotherapy-induced nausea and vomiting.

A retrospective chart review by Okamoto H et al. answered the question of whether olanzapine was beneficial for CAS patients without the presence of nausea and vomiting [34]. The investigators reviewed the inpatient data records of 951 patients served by a palliative care team at Chiba University Hospital in Japan from 2008 to 2016. Records of food intake three days before and after the initiation of olanzapine documented by nurses were retrieved and analyzed. Inclusion criteria were cancer diagnosis, prescribed olanzapine, and >grade 1 appetite loss in the Common Terminology Criteria for Adverse Events (CTCAE). Those who had >Grade 1 somnolence in CTCAE, intestinal obstruction, and newly started or newly increased corticosteroid therapy were excluded. A subgroup analysis was conducted excluding those who had >Grade 1 nausea or vomiting or who were on newly started or newly increased doses of antiemetic medications. A total of 80 cases were identified representing a variety of cancer types. The average age of the cohort was 60.4 years old (standard deviation 15.7 years). Among those patients, the average olanzapine dose was 2.28 ± 0.87 mg per day (mean ± SD) and the range was 1.5 mg to 5 mg per day. The result shows that food intake was increased by 149% on average after starting olanzapine compared to before (*p* < 0.0001). Additionally, the food intake was significantly higher after starting olanzapine compared to before in the 40 nausea-free patients, averaging a 143% increase in food intake (*p* < 0.001). One limitation of this study was the retrospective nature, small sample size, and the short duration of observation (3 days). However, this study provided insights into olanzapine’s effect on food intake in those without nausea and vomiting and corticosteroid therapy. It also provided knowledge that the action of olanzapine on food intake can be observed within 3 days.

Okamoto et al.’s finding was confirmed by a recent randomized placebo-control trial by Navari RM et al. [35]. The study investigated the impact of olanzapine in a cohort of advanced cancer patients with chronic nausea and vomiting who were not on chemotherapy or radiotherapy. Thirty (30) participants received either 5 mg of olanzapine orally once daily (*n* = 15) or a placebo for 7 days (*n* = 15), and their level of nausea, vomiting, appetite, sedation, fatigue, pain, and well-being were assessed using a numeric rating scale. Alternate antiemetics were allowed if nausea and vomiting became uncontrolled. Both clinicians and patients were blinded to study drug allocation. Only one participant dropped out of the study due to nausea, but all thirty participants were analyzed (intention-to-treat). The study found that the olanzapine arm was associated with significantly less nausea (*p* < 0.001), fewer episodes of vomiting (*p* = 0.001), better appetite (*p* < 0.001), less fatigue (*p* = 0.004), and better well-being (*p* < 0.001) compared to the placebo. One strength of this study was the randomized controlled design even though the small sample size was small.

Dev R et al. described three case reports using olanzapine in a variety of combinations for off-labeled indications, including refractory nausea, anxiety, insomnia, depression, and poor appetite [36]. In one of the cases, the patient presented with anxiety, depression, and profound fatigue on escitalopram and alprazolam. Alprazolam was tapered over 2 weeks, and 5 mg of olanzapine every 8 h as needed was initiated for anxiety and insomnia. The patient reported reduced anxiety and tolerated the drug well at one month, maintaining good control with alprazolam tapered off and continued escitalopram and olanzapine (2.5 mg during the day and 5 mg at bedtime). Another case reported was a patient who was prescribed 4 mg of dexamethasone twice daily and 5 mg of olanzapine at bedtime for fatigue, depression, poor appetite, and sleep problems. Five days after starting olanzapine, the patient developed confusion and was diagnosed with hyperammonemia [42]. Dev R et al.’s case reports provided detailed descriptions of three different scenarios where olanzapine was used with cancer patients for symptom control, highlighting the importance of recognizing olanzapine’s pharmacological action on multiple receptor sites with potential antiemetic, appetite-stimulating, augmentative anti-depressive, and sedative effects [43,44].

## 4. Discussion

a. What doses have been studied and are appropriate for this indication?

Naing A. et al. and Braiteh F et al. used dose escalation (“titration”) based on individual patients’ tolerability of the drug, and Navari RM et al. and Sandhya L et al. used a low dose (5 mg/day and 2.5 mg/day, respectively) for all enrolled participants [30,31,32,33]. One rationale for dose titration is the propensity of olanzapine to cause sedation and drowsiness if the patient starts on a higher dosage. Sedation is a common side effect of olanzapine (up to 30%) and patients may develop tolerance to this side effect with time [45]. Initiating the drug slowly allows the patient to develop tolerance and experience fewer side effects. A common titration schedule starts at 2.5 mg every day and increases in 2.5–5 mg increments, up to 15 mg [30] to 20 mg every day [32]. A randomized double-blind placebo-controlled study (*n* = 124) showed that 2.5 mg of olanzapine once daily for 12 weeks resulted in significant weight gain and improved appetite compared to a placebo [38]. Therefore, there is evidence to believe that a low dose of olanzapine is sufficient for cancer cachexia without the need to titrate to a target dose. However, if dose escalation is warranted, the dose should be titrated in small increments to reduce side effects. Olanzapine is available in tablets, oral disintegrating tablets, and injection formulations; most studies used the oral tablet formulation [30,32,35,38], and one study used olanzapine powder (available in Japan) and tablets [34]. Olanzapine is well absorbed, and the tablet and oral disintegrating tablets are bioequivalent to oral tablet formulation [46]. Therefore, for those with difficulty in swallowing, the oral disintegrating formulation can theoretically be interchanged with the oral tablet formulation [47].

b. Who will be a candidate for low-dose olanzapine?

Based on our review, current original research studies were limited to adults ≥ 18 years old who had to be able to swallow tablets. There is no supportive evidence on the use of olanzapine for pediatric CAS patients. We noticed that the definitions of CAS varied (e.g., 10% loss over 6 months [32] versus ≥ 5% weight loss [35]) among studies, and we suggest that utilizing standardized diagnostic criteria for cancer cachexia in future studies and practice would be helpful [3]. We noted that two studies included participants on concurrent chemotherapy [35,48] and one study excluded them [35]. Those who are on emetogenic chemotherapy will likely have added benefits, since olanzapine is an antiemetic agent for chemotherapy-induced nausea and vomiting [49,50]. In a systemic review by Ji M et al. investigating the changes in quality of life among patients who received olanzapine, five out of six studies enrolled participants on moderately or highly emetogenic chemotherapy [48]. While all studies in the review involved advanced cancer patients, olanzapine for CAS has been studied in stage III and IV gastric, lung, hepatopancreatic, and colon cancer in prospective randomized studies [33,35].

c. What are the expected therapeutic outcomes and duration of treatment, and how do we monitor them?

The most common indicator in monitoring the therapeutic effect of olanzapine in the reviewed studies was weight. We noted some variation in the classification of a clinically significant response: one study used a ≥5% weight gain as the primary endpoint [31], one study used >5% weight gain, and one study used weight slope [32]. Most studies measured patient-reported appetite and nausea level using the visual analog scale, and one study defined a +3 in visual analog scale as a clinically significant change [35]. The increase in food intake can be observed within days [34] after initiating olanzapine, and the positive impact in weight gain can be seen up to 12 weeks [33].

d. Toxicities and Monitoring

Olanzapine can cause a range of side effects, including extrapyramidal symptoms, hyperglycemia, dyslipidemia, postural hypotension, QTc prolongation, anticholinergic side effects, priapism, sedation, seizures, cerebrovascular events, blood dyscrasias, and neuroleptic malignant syndrome [45]. Most side effects are dose-dependent, meaning a higher dose will be associated with higher risk. Interestingly, weight gain, a side effect of olanzapine that we are utilizing for CAS patients, does not appear to be dose-dependent and is commonly reported to be early-onset with rapid increase and plateau over time [45]. In older adults, the presence of renal and hepatic impairment increases the risk of developing adverse events of antipsychotics [40]. Olanzapine, being an atypical antipsychotic, is associated with less EPS compared to conventional antipsychotics [51], as well as being prolactin-sparing (exerts minimal effects on prolactin levels) [52]. Olanzapine has a higher risk of causing hyperglycemia and increased levels of cholesterol and triglycerides than other atypical antipsychotics and conventional antipsychotics [45]. A meta-analysis found increased mortality for patients with dementia after receiving atypical antipsychotics compared to the placebo (odds ratio, 1.536, 95% C.I. 1.028–2.296; *p* = 0.036), and the estimated odds ratio was highest for olanzapine (OR 1.919) compared to other atypical agents [53].

Routine monitoring of olanzapine includes mental status and signs and symptoms of EPS. The monitoring parameters for patients taking olanzapine for CAS should require special consideration by developing a patient-centered care plan that aligns the goals of care and the assessments needed to achieve clinical outcomes. As the cancer progresses, the goals of care may shift from curative to non-curative symptom control, and the laboratory and weight monitoring frequency may need to be re-evaluated to reduce invasive monitoring and transportation time to healthcare facilities. Patients and caregivers should be made aware of symptoms they can monitor at home and report to clinicians, such as food intake, involuntary muscle contraction (acute dystonia), facial expression (tardive dyskinesia), slow and rigid gait (pseudoparkinsonism), restlessness (akathisia), and a change in mentation [46].

e. Applications to pharmacy and pharmacy education

The therapeutic use of olanzapine is typically taught as part of the schizophrenia lectures in the pharmacy curriculum. This article highlights an example of using olanzapine’s effects on weight gain for CAS patients where no other FDA-approved medications exist for this indication. It is important for the pharmacist, as a drug expert, to recognize the dose range of olanzapine that has shown benefits for CAS and the monitoring parameters involved, especially for those with organ impairment who are vulnerable to experiencing the multitude of side effects associated with olanzapine. Clinical pharmacists can serve an integral role in the interdisciplinary team in palliative care by providing drug information and monitoring therapeutic efficacy and toxicity.

## 5. Conclusions

Our literature review provided detailed information about the therapeutics of olanzapine for CAS patients in support of the ASCO recommendation. Our literature search showed that low-dose olanzapine (2.5–5 mg once daily) may provide benefits in weight gain for patients with cancer anorexia-cachexia syndrome. Olanzapine doses as low as 2.5 mg once daily have been shown to produce significant weight gain in a randomized controlled trial. The therapeutic responses that can be observed early after initiating the drug are increased appetite, improved sleep, and reduced nausea. Most adverse events associated with olanzapine are dose-dependent; thus, using low-dose olanzapine is associated with a relatively low incidence of adverse events in clinical studies. Clinicians should discuss the common adverse events and the symptoms to monitor with the patient and caregiver before starting olanzapine for CAS.

## Figures and Tables

**Table 1:** Primary literature related to Olanzapine for CAS.

Authors (Year)	Study Design	Olanzapine Regimen	Outcomes	Results
Braiteh F et al. (2008) [30]	Prospective open-label (*n* = 14)	Initial dose of 2.5 mg via mouth once daily and titrate up to 15 mg once daily	Weight, Mini-Nutritional Assessment (MNA), Karnofsky performance scores, 6-min walking test, Edmonton Symptoms Assessment Scale (ESAS), cytokine, leptin, ghrelin, adiponectin levels	A total of 6 out of 14 participants experienced weight stabilization or gain at 2 weeks. Improvements in physical performance and ESAS were observed.
Navari RM et al. (2010) [31]	Prospective randomized study (*n* = 80)	800 mg of megestrol acetate (MA) via mouth per day vs. 5 mg of MA plus olanzapine via mouth once daily for 8 weeks	>5% weight gain, appetite and nausea, and quality of life improvement	MA plus olanzapine group was associated with higher rates of >5% weight gain, appetite, nausea, and quality of life improvement compared to MA only. No grade III or IV toxicities were reported.
Abdelrahim M et al. (2013) [37] Naing A et al. (2015) [32]	Prospective open-label (*n* = 39)	An initial dose of 2.5 mg via mouth once daily, using a 3 + 3 design, and up to 20 mg once daily	Cytokine, leptin, growth hormone, interleukin-6, ghrelin levels; change in weight	A total of 4 out of 39 participants experienced non-dose limiting toxicities. Changes in weight and cytokines were not conclusive pre- and post-olanzapine.
Singuluri SL et al. (2022) [38] Sandhya L et al. (2023) [33]	Randomized double-blind placebo-controlled study (*n* = 124)	2.5 mg of olanzapine once daily for 12 weeks or placebo	>5% weight gain, appetite, and quality of life improvement	Patients in the olanzapine arm were significantly associated with weight gain >5%, appetite, and quality of life improvement compared to the placebo group.
Okamoto H et al. (2019) [34]	Retrospective chart review (*n* = 80)	Olanzapine once daily at night between 6 pm and midnight, 1.5–5 mg per day (mean 2.28 mg)	Food intake according to nursing records 3 days before and 3 days after starting olanzapine	Increased food intake was observed after starting olanzapine.
Navari RM et al. (2020) [35]	Randomized controlled trial	5 mg of olanzapine once daily or placebo for 7 days	Change in nausea and appetite measured using numeric rating scores	Patients who received olanzapine experienced significantly less nausea and vomiting and improved appetite and well-being.
Dev R et al. (2022) [36]	Case reports (*n* = 3)	2.5 mg via mouth every 12 h, as needed, or 5 mg at night for nausea, anorexia, anxiety, and insomnia	Individual reports of symptom control in nausea, appetite, sleep, anxiety	Low-dose olanzapine was useful in relieving nausea, appetite, and sleep. One patient presented with hyperammonemia 5 days after initiation of 5 mg of olanzapine, and mentation was resolved after drug discontinuation and treatment.

## Data Availability

Not applicable.
